# The Ca^2+^-Regulation of the Mitochondrial External NADPH Dehydrogenase in Plants Is Controlled by Cytosolic pH

**DOI:** 10.1371/journal.pone.0139224

**Published:** 2015-09-28

**Authors:** Meng-Shu Hao, Anna M. Jensen, Ann-Sofie Boquist, Yun-Jun Liu, Allan G. Rasmusson

**Affiliations:** 1 Department of Biology, Lund University, Lund, Sweden; 2 Department of Forestry and Wood Technology, Linnaeus University, Växjö, Sweden; 3 Institute of Crop Science, Chinese Academy of Agricultural Sciences, Beijing, China; Universidade Federal de Vicosa, BRAZIL

## Abstract

NADPH is a key reductant carrier that maintains internal redox and antioxidant status, and that links biosynthetic, catabolic and signalling pathways. Plants have a mitochondrial external NADPH oxidation pathway, which depends on Ca^2+^ and pH in vitro, but concentrations of Ca^2+^ needed are not known. We have determined the K_0.5_(Ca^2+^) of the external NADPH dehydrogenase from *Solanum tuberosum* mitochondria and membranes of *E*. *coli* expressing *Arabidopsis thaliana* NDB1 over the physiological pH range using O_2_ and decylubiquinone as electron acceptors. The K_0.5_(Ca^2+^) of NADPH oxidation was generally higher than for NADH oxidation, and unlike the latter, it depended on pH. At pH 7.5, K_0.5_(Ca^2+^) for NADPH oxidation was high (≈100 μM), yet 20-fold lower K_0.5_(Ca^2+^) values were determined at pH 6.8. Lower K_0.5_(Ca^2+^) values were observed with decylubiquinone than with O_2_ as terminal electron acceptor. NADPH oxidation responded to changes in Ca^2+^ concentrations more rapidly than NADH oxidation did. Thus, cytosolic acidification is an important activator of external NADPH oxidation, by decreasing the Ca^2+^-requirements for NDB1. The results are discussed in relation to the present knowledge on how whole cell NADPH redox homeostasis is affected in plants modified for the *NDB1* gene.

## Introduction

Ca^2+^ has a central function in signalling in plant cells, being regulatory in several intrinsic and extrinsic conditions including abiotic and biotic stress responses, stomatal closure, tip growth and photoreceptor signalling [1]. The cytosolic Ca^2+^ concentration will in an unstimulated or “resting” cell reside at a low level of around 100 nM. However, in response to stimuli, oscillatory increases in the cytosolic Ca^2+^ concentration occur. The amplitude and period depend on stimuli and cell type, each combination having its specific Ca^2+^ signature [2]. Cytosolic Ca^2+^ concentrations as high as 5 μM or higher have been recorded in response to external stress [3]. In addition to major effects on gene expression [4], Ca^2+^ also interacts directly with the cytosolic cellular redox metabolism at several points, *e*.*g*. by binding EF-hand domains that are present in many plant enzymes [5]. A direct interaction with redox enzymes in metabolic pathways is for example seen in the Ca^2+^-activation of the stress-associated gamma-aminobutyrate shunt [6]. Further, the plant respiratory burst oxidase protein AtRBOHC respond to reactive oxygen species (ROS) by oxidising cytosolic NADPH and produce apoplastic superoxide that opens Ca^2+^-channels that, in turn, leads to a Ca^2+^-activation of the same oxidase [7]. Roles in NADPH redox homeostasis have also been suggested for Ca^2+^ via its interaction with mitochondrial Ca^2+^-dependent enzymes like NAD kinase and external NAD(P)H dehydrogenases (DHs) [8,9].

Mitochondria generate ATP and carbon compounds for cellular reactions. Further, plant mitochondria are also functionally involved in photosynthetic metabolism [10], nitrogen metabolism [11], ROS signalling [8], plant stress [12,13], reactive oxygen sensing and balancing [14], and under certain conditions also heat production [15]. The multiple roles of mitochondria in plants are reflected in a highly complex electron transport chain (ETC). The ETC is located in the inner mitochondrial membrane and contains four integral protein complexes (I-IV) as in most eukaryotic cells. Compared to mammals, the ETC in plants is more complex due to the presence of alternative electron transport enzymes, especially type II NAD(P)H DHs and alternative oxidase (AOX). ATP synthesis is derived from the electrochemical proton gradient across the inner mitochondrial membrane, which couples the transfer of electrons by complexes I, III and IV to the ATP synthase. However, alternative electron transport is not linked to proton transport and ATP formation, and can instead allow oxidation of redox compounds like NAD(P)H independent of the cellular ATP status [16–18]. The AOX pathway has been shown to have a particular role under conditions of cellular oxidative stress caused by external plant stress or mitochondrial dysfunction, and involving enhanced ROS levels [16]. Less is known about the physiological function of the type II NAD(P)H DHs, but modification of enzyme levels in transgenic plants have revealed the ability of the individual enzymes to modify the total cellar NAD(P)H reduction level, in turn affecting metabolism and development [19–22].

In plant mitochondria, type II NAD(P)H DHs are located both on the inner and outer surface of the inner mitochondrial membrane [9]. The oxidation of cytosolic NADH and NADPH are catalysed by separate external DHs [23–25]. Ca^2+^-dependence has been shown for NADH oxidation, though the Ca^2+^-dependence varies with the plant material analysed [26,27]. In Jerusalem artichoke (*Helianthus tuberosus)* mitochondria the Ca^2+^ concentration needed for halfway stimulation (K_0.5_(Ca^2+^)) of NADH oxidation was shown to be below 1 μM and was 5 times lower in the presence of polyamines [28,29]. However, a similar investigation has not been reported for NADPH. External NADPH oxidation activity is Ca^2+^-dependent at neutral pH, while it is Ca^2+^-independent under more acidic conditions [30].

External NAD(P)H DHs are encoded by nuclear *NDB* genes, which are characterised by an internal domain containing an EF-hand motif and an EF-hand like motif [19,31]. This domain is absent in most type II NAD(P)H DHs. Potato NDB1 (StNDB1) was shown to be an external Ca^2+^-dependent NADPH DHs [23]. In *Arabidopsis thaliana*, AtNDB1 is specific for NADPH and activated by Ca^2+^ via binding to the EF-hand domain, whereas the AtNDB2 and AtNDB4 are Ca^2+^-stimulated and Ca^2+^-independent NADH DHs, respectively [32]. Similar substrate specificities and pH dependencies were observed with oxygen as final acceptor (i.e. via the natural ubiquinone) and using the decylubiquinone analogue [32].

The abundance of the mitochondrial external NADPH DH affects the whole cell NADPH reduction level in *A*. *thaliana* and *Nicotiana sylvestris* and the activity *in vitro* is affected by both Ca^2+^ and pH [19,21]. However, the Ca^2+^ treshold concentrations needed for activation are not known nor if Ca^2+^ and pH cooperate in establishing the *in vivo* activity. We have therefore determined the Ca^2+^ requirement for activation of the external NADPH DH in potato mitochondria and in membranes from *E*. *coli* expressing AtNDB1, using different ubiquinone acceptors. We have also determined how the Ca^2+^ requirement varies with pH within the physiological pH range, observing clear indications that cytosolic acidification is a critical activator of the NADPH DH activity due to a pH regulation of the Ca^2+^ activation.

## Materials and Methods

### Preparation of mitochondria and recombinant *A*. *thaliana* NDB1

Intact mitochondria were isolated from potato tubers (*Solanum tuberosum* L. cv. Bintje) and *N*. *sylvestris* leaves according to published procedures [19,33]. The transgenic *N*. *sylvestris* lines overexpressing and sense-suppressed for StNDB1 have been previously described [34,35]. Membranes containing AtNDB1 were produced and isolated from *E*. *coli* carrying the recombined plasmid pET-T7NDB1 as previously described [32]. Isolated mitochondria and *E*. *coli* membranes were supplemented with dimethyl sulfoxide to a final concentration of 5% (v/v), frozen in liquid nitrogen, and stored at -80°C. The protein contents of the mitochondrial samples were determined by the BCA method (Sigma, St. Louis, MO).

### Mitochondrial integrity assay

The integrity of the mitochondrial inner membrane was determined as the latency for Ca^2+^-independent NADH oxidation, measured spectrophotometrically (A_340_-A_400_) in an Olis-Aminco DW2a. NADH consumption was measured in a reaction mix containing 50 mM sucrose, 1 mM EGTA, 100 mM KCl, 20 mM MOPS/KOH (pH 7.2), 0.1 mM NADH and approximately 0.1 mg mitochondrial protein. After determining the initial rate, the maximum rate was determined by adding the channel-forming peptide alamethicin (Alm) [36] to a final concentration of 20 μg/ml. The latency was calculated as (Activity_+Alm−_Activity_-Alm_)/ Activity_+Alm_ × 100%.

### Assay media

NAD(P)H oxidation activities were determined using several media. Medium 1 contained 50 mM sucrose, 100 mM KCl and 20 mM of a zwitterionic buffer (pH set with KOH). The buffers used were MES, PIPES, MOPS and HEPES for pH 6.0, 6.8, 7.2 and 7.5, respectively. Medium 1+Mg^2+^ was additionally supplemented with 2.5 mM MgCl_2_. Medium 2 was similar to Medium 1 but contained a buffer made by mixing the medium supplemented with 20 mM BisTris, 20 mM HCl with the medium supplemented with 20 mM triethanolamine, 20 mM KCl to a final pH of 7.2 [37]. The total concentrations of EGTA are specified in the legend of each data presentation.

For determination of the concentration of Ca^2+^ allowing half-maximum activity (K_0.5_(Ca^2+^)), we produced series of activity assay media containing Ca^2+^/EGTA buffers with known free Ca^2+^ concentrations by adding a Ca^2+^ stock solution (by weight) to media with nominal EGTA concentration of 1 mM. After re-adjusting the pH with KOH, the media aliquots were measured with a Ca^2+^-selective electrode (Radiometer, Copenhagen, Denmark). Apparent Ca-EGTA stability constants, actual EGTA concentrations, and free Ca^2+^ concentrations of the media were calculated as previously described [38]. K_0.5_(Ca^2+^) were calculated as-log[Ca^2+^] (pCa) and converted to μM where denoted. A representative example of a Ca^2+^/EGTA buffer curve is given in S1 Fig.

### Activity measurements

O_2_ consumption was measured in Medium 1 in an O_2_-electrode (Rank Brothers, UK), in a total volume of 1 ml at 25°C. The electrode was calibrated using O_2_-saturated water. Each assay contained 0.4 μM carbonyl cyanide *p*-trifluoromethoxy phenylhydrazone (FCCP) and 1 mM NAD(P)H, and was started with the addition of 20 μg mitochondrial protein. For easier comparison, the activities were recalculated to NAD(P)H oxidation using a factor of 2 NAD(P)H/O_2_.

NAD(P)H consumption was measured by dual wavelength spectrophotometry as A_340_-A_400_ in an Olis Aminco DW2a spectrophotometer (5 nm slit) at 22°C in a total volume of 1 ml, unless otherwise specified. NAD(P)H was used at 80 μM. For measurements with O_2_ as final electron acceptor, 0.4 μM FCCP was added, whereas assays with decylubiquinone (DcQ) as electron acceptor contained 1 mM KCN and 20 μM DcQ. Reactions were started with the addition of 0.01–0.04 mg mitochondrial protein or 0.02–0.03 mg *E*. *coli* membrane protein. Determinations of rate changes after shifts in Ca^2+^ concentrations were carried out in the same way, but in a 2 ml volume with constant stirring by a magnetic micro stir bar.

### Data analysis

For analysis of NAD(P)H oxidations activities at different free pCa, data from all biological replicates were pooled and fitted using sigmoidal curve fit in Kaleidagraph 4.1.3 (Synergy Software, Reading, PA, USA). From the curve fits, values and standard errors for maximum rate and K_0.5_(Ca^2+^) were extracted. R for the curve fits was on average 0.97 and in each case at least 0.90. The equation used for the curve fit was

$$v\left(pCa\right)=a+\frac{b-a}{1+{\left(\frac{pCa}{c}\right)}^{d}}$$

where *v* is the rate, *a* and *b* are minimum and maximum rates, respectively, *c* is –log (K_0.5_(Ca^2+^)) and *d* is a constant.

For determining the rate of activity changes after a rapid decrease in Ca^2+^ concentration, curve fitting was carried out using a least square exponential decay function in Kaleidagraph 4.1.3, and activity half-life values were calculated separately for each biological replicate before averaging. R was for each curve fit at least 0.97. The equation used for the curve fits was

$$v\left(t\right)=a+\left(b-a\right)e^{-ct}$$

where *v* is the rate, *a* is the minimal rate, *b* is the maximum rate, *c* the rate constant and *t* the time. Half-life (*t*_*1/2*_) was then calculated based on

$$v\left(t_{\frac{1}{2}}\right)=\frac{b-a}{2}+a$$

One-way ANOVA was carried out using the Tukey posthoc test in Kaleidagraph 4.1.3.

## Results

### NADH DH latency of potato mitochondria

The inner membrane integrity of isolated mitochondria from potato tubers was tested as the Alm permeabilisation-dependence of EGTA-insensitive NADH oxidation, the activity of which depends on the access of NADH to membrane-bound DHs oriented only towards the matrix. We observed a high maximum activity of 480 ± 85 nmol min^-1^ mg protein^-1^ (mean ± SE) and an Alm latency of 94 ± 1.5%. This indicates that the inner membranes of the mitochondria were practically impermeable to reduced pyridine nucleotides, and that a measured NADPH oxidation in the absence of Alm would only be due to the activity of the external NADPH DH.

### Potato external NAD(P)H oxidation is Ca^2+^-dependent in the neutral pH range

**Fig 1:**
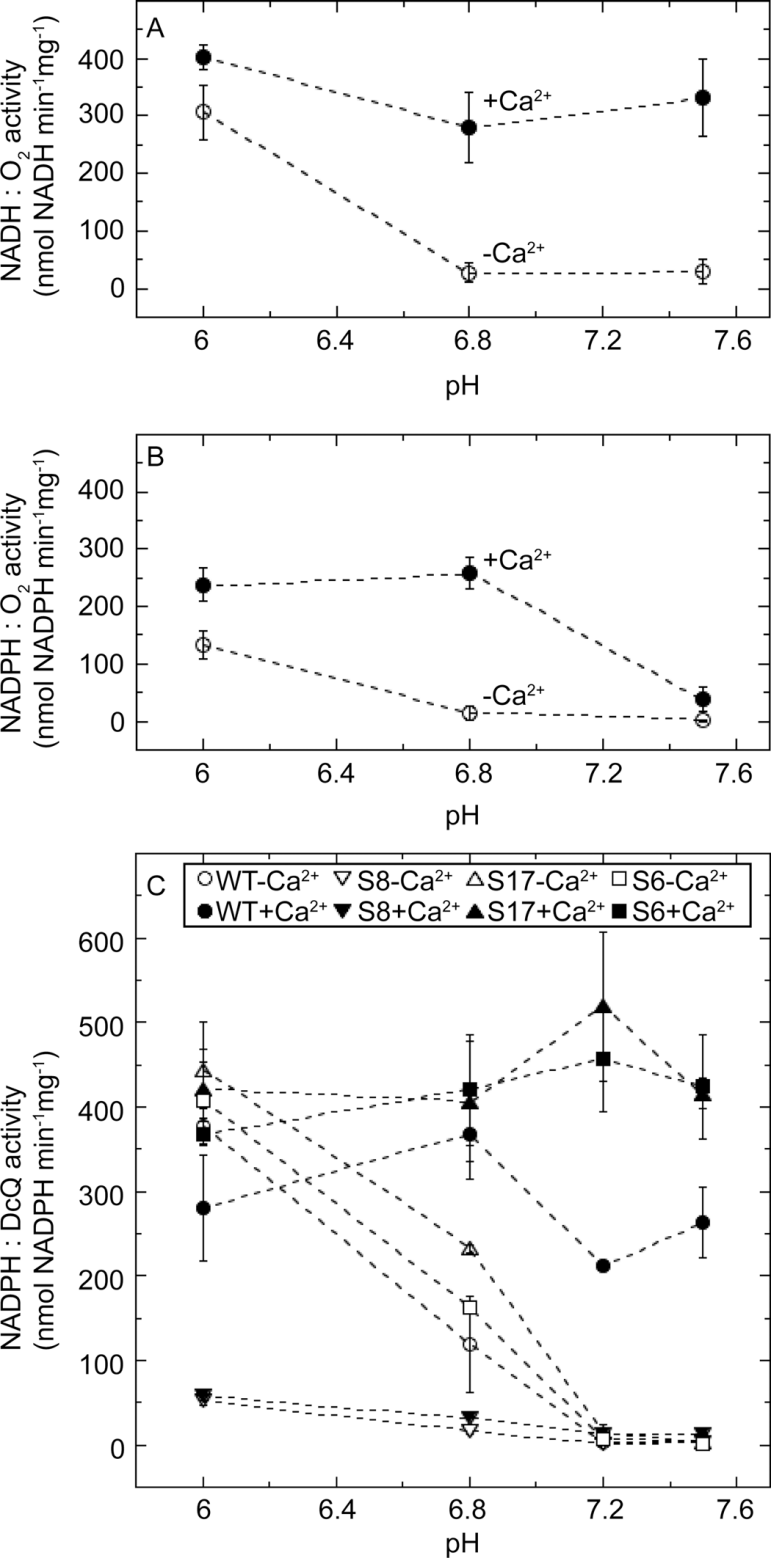
Ca^2+^ dependence of NAD(P)H oxidation in mitochondria at different pH values. (A) NADH and (B) NADPH oxidation activity of potato tuber mitochondria were measured at pH 6.0–7.5 as O_2_ consumption, using 1 mM NAD(P)H as substrate. (C) NADPH oxidation with DcQ as final electron acceptor in mitochondria from *N*. *sylvestris* expressing StNDB1 was measured at pH 6.0–7.5. Closed and open symbols denote presence and absence of 1 mM free Ca^2+^, respectively. In (C), wildtype (WT) *N*. *sylvestris*, the overexpression lines S17 and S6, and the suppression line S8 are denoted as circles, triangles, squares and inverted triangles, respectively. Data displays average for 2–3 independent mitochondrial preparations, with error bars denoting SD in (A) and (B) and SE in (C).

External NAD(P)H oxidation is Ca^2+^-dependent in the neutral pH range but not at low pH in spinach and *H*. *tuberosus* mitochondria [30,39]. In potato (Fig 1A), the NADH oxidation activity was 5–8 fold higher in the presence of Ca^2+^ than in its absence at pH 6.8 and pH 7.5, whereas the activity at pH 6 was Ca^2+^-independent. In a similar way, NADPH oxidation strongly depended on the presence of Ca^2+^ at pH 6.8 and 7.5, but not at pH 6.0 (Fig 1B). However, unlike NADH oxidation, NADPH oxidation decreased drastically from pH 6.8 to pH 7.5.

To determine if other enzymes than type II NAD(P)H dehydrogenases may contribute to mitochondrial NADPH oxidation at low pH, we investigated mitochondria from *N*. *sylvestris* lines transformed with a sense construct for *StNDB1* expression [19,20,23]. The overexpression of StNDB1 in lines S6 and S17 lead to higher NADPH:DcQ activity in the presence of Ca^2+^ at pH 7.2 and 7.5 (Fig 1C). However, the joint sense-suppression of *StNDB1* and the endogenous *NsNDB1* in line S8 [19] abolished both Ca^2+^-dependent and Ca^2+^-independent NADPH:DcQ activity at all pHs investigated. Thus, the external NADPH oxidation activity is dependent on the presence of an NDB1-type enzyme(s) across the whole pH range.

Alignment of all NDB proteins in the fully sequenced genomes of potato and *A*. *thaliana* (S2 Fig) showed that StNDB1 and AtNDB1 are the sole NDB-proteins that carry the active site sequence motif previously characterised for the NADPH-specific type II DHs [23,32]. Thus, the external NADPH oxidation measured in isolated potato mitochondria relates solely to the StNDB1 protein.

### The Ca^2+^-dependence of mitochondrial NADPH oxidation is affected by pH

**Fig 2:**
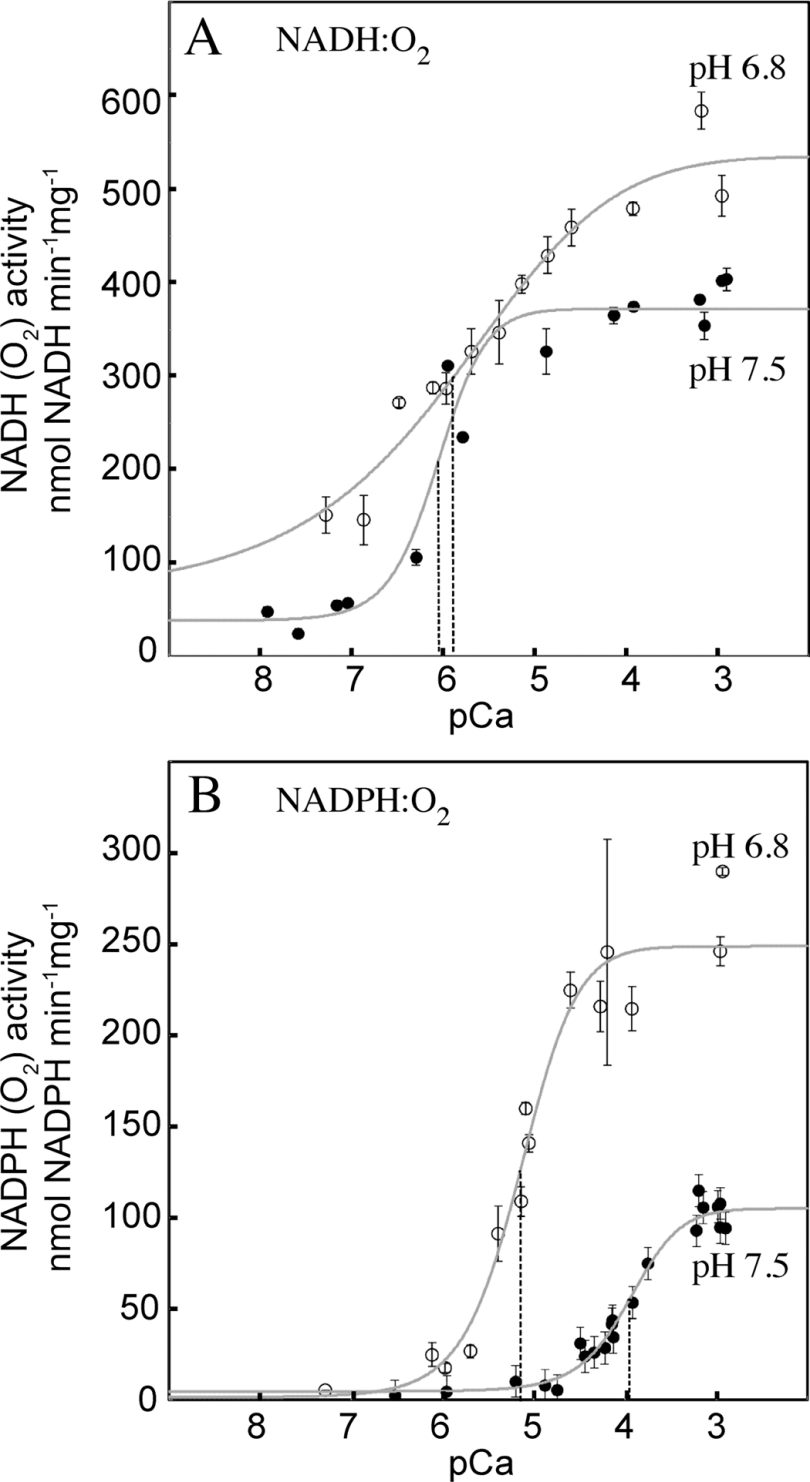
Ca^2+^ requirements of external NAD(P)H oxidation in potato mitochondria at pH 6.8 and 7.5. Oxidation of 1 mM NADH (A) and NADPH (B) was measured as oxygen consumption in Medium 1 at pH 6.8 (open symbols) and pH 7.5 (closed symbols) with 1 mM total EGTA and defined free Ca^2+^ concentrations. The graphs show pooled data points from two (A) and three (B) independent mitochondrial preparations and error bars indicate SD for 2–3 measurements per point. The sigmoidal curve fits are denoted as continuous grey lines. The K_0.5_(Ca^2+^) value for each curve is denoted with a vertical line.

External NADH oxidation in *H*. *tuberosus* mitochondria is halfway activated by Ca^2+^ (K_0.5_(Ca^2+^)) at 0.2–1 μM at pH 7.2 [28,29]. To determine K_0.5_(Ca^2+^) also for NADPH oxidation and to see if pH affects the K_0.5_(Ca^2+^) in the neutral range, we prepared series of media with defined free [Ca^2+^] at pH 6.8 and 7.5 (S1 Fig). NAD(P)H oxidation to O_2_ at different concentrations of Ca^2+^ is shown in Fig 2. NADH oxidation was halfway activated at pCa values of 5.68±0.19 and 6.07±0.10 at pH 6.8 and 7.5, respectively (± SE)). The concentration dependence is consistent with the previous K_0.5_(Ca^2+^) determinations at pH 7.2 [28,29] and further indicates that K_0.5_(Ca^2+^) for NADH oxidation is affected little by pH. In contrast, NADPH oxidation was halfway activated at pCa values of 5.19±0.12 and 3.95±0.05 at pH 6.8 and 7.5, respectively, corresponding to a 19-fold difference in K_0.5_(Ca^2+^) between the two pH values. Thus, NADH oxidation in potato mitochondria is activated by low Ca^2+^ concentrations, but NADPH oxidation demands higher Ca^2+^ concentrations, which at pH 7.5 is outside the physiological range. The large variation in K_0.5_(Ca^2+^) observed for NADPH oxidation indicates that the Ca^2+^ activation of NDB1 strongly depends on the pH.

### The Ca^2+^-dependence varies with the quinone acceptor

**Fig 3:**
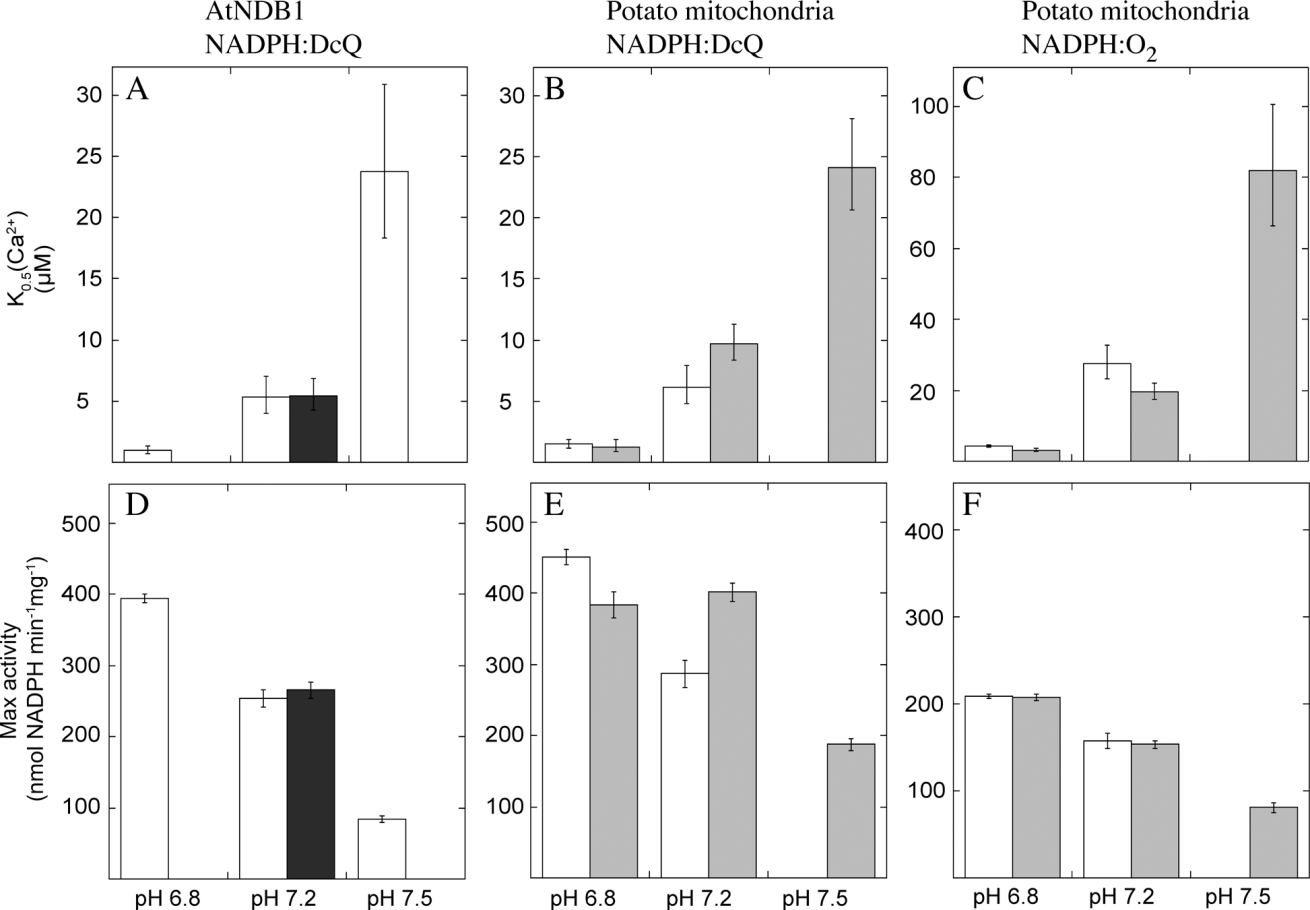
Ca^2+^ dependence of StNDB1 and AtNDB1 using different electron acceptors and media. (A) K_0.5_(Ca^2+^) of AtNDB1 NADPH oxidation with DcQ as final electron acceptor at different pH in Medium 1 (light grey) and in Medium 2 (dark grey). (B) K_0.5_(Ca^2+^) for the NADPH:DcQ activity in potato mitochondria at various pHs in the absence (Medium 1; light grey) and presence of 2.5 mM MgCl_2_ (Medium 1+Mg^2+^; intermediate grey). (C) K_0.5_(Ca^2+^) for the NADPH:O_2_ activity in potato mitochondria at various pHs in the absence (Medium 1) and presence of 2.5 mM MgCl_2_ (Medium 1+Mg^2+^). NADPH oxidation was measured spectrophotometrically using 80 μM NADPH as substrate. Graphs (D-F) show the maximum activities corresponding to the data in (A-C), respectively. The error bars indicate SE for the sigmoidal curve fits, each being made using pooled data from 2–3 independent mitochondrial or *E*. *coli* preparations.

To investigate the interaction of Ca^2+^ and pH regulation in more detail we measured Ca^2+^ activation of the NADPH oxidation activity using different electron acceptors and media by dual wavelength spectrophotometry. The StNDB1 enzyme was assayed as present in isolated potato mitochondria and AtNDB1 in membranes isolated from the *E*. *coli* MWC008(DE3) double mutant lacking NADH DHs and expressing recombinant AtNDB1 [32]. For NADPH oxidation to the short-chained ubiquinone analogue DcQ by recombinant AtNDB1, the K_0.5_(Ca^2+^) was lowest at pH 6.8 (0.95 μM) and increased at higher pHs, up to 24 μM at pH 7.5 (Fig 3A). The K_0.5_(Ca^2+^) at pH 7.2 was similar independent of the buffering compound used, being 5.3–5.4 μM in both a zwitterionic buffer (MOPS/KOH; Medium 1) and a BisTris/triethanol amine-based buffer system (Medium 2).

The K_0.5_(Ca^2+^) for NADPH oxidation to DcQ by potato mitochondria was 1.5 and 6.2 μM at pH 6.8 and 7.2, respectively (Fig 3B), i.e. similar to the corresponding values observed for AtNDB1 (Fig 3A). Using the same assay method, the K_0.5_(Ca^2+^) for NADPH oxidation to O_2_ was, 4.4 and 27.5 μM at pH 6.8 and 7.2, respectively (Fig 3C), which is 3–4 times greater than the corresponding values with DcQ (Fig 3B). With the additional inclusion of 2.5 mM Mg^2+^ to the media similar observations were made at pH 6.8 and 7.2 (Fig 3B and 3C). At pH 7.5 in the presence of Mg^2+^, high K_0.5_(Ca^2+^) values were observed, being 24.1 and 81.8 μM, with DcQ and O_2_ as final electron acceptors, respectively (Fig 3B and 3C). For all conditions, the values of maximum NADPH oxidation were decreased (50–75%) with increasing pH over the measured range (Fig 3D–3F). The results thus show that the K_0.5_(Ca^2+^) values using DcQ as acceptor are highly similar in potato and *A*. *thaliana*, and substantially lower than what is observed for potato mitochondria using O_2_ as acceptor.

### External NADPH oxidation responds rapidly to Ca^2+^ depletion

**Fig 4:**
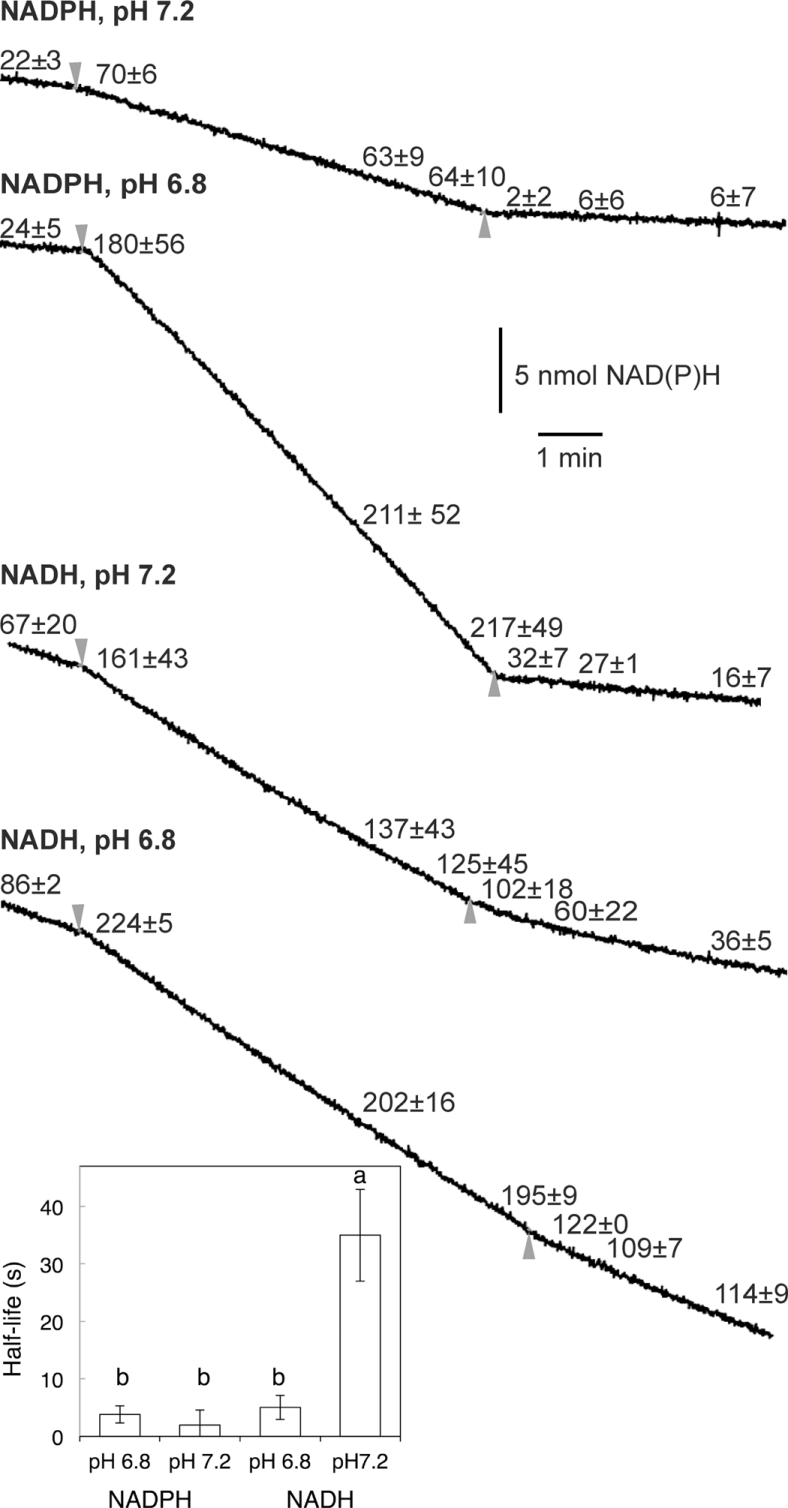
Responsiveness of NAD(P)H oxidation to changes in Ca^2+^ concentration. Ca^2+^ activation and EGTA inactivation rates for external NAD(P)H oxidation to O_2_ by potato mitochondria were determined by dual-wavelength spectroscopy at pH 6.8 and pH 7.2. The assays were made in 2 ml Medium 1 initially containing 50 μM EGTA. Inverted triangles denote the addition of CaCl_2_ to a total concentration of 90 μM, giving a free Ca^2+^ concentration of approximately 40 μM, irrespective of pH. Upright triangles denote the addition of EGTA to a total concentration of 250 μM, leading to a free Ca^2+^ concentration of approximately 0.1 and 0.5 μM at pH 7.2 and 6.8, respectively. The values along the curves display the activities 15 s, 1 min and 4 min after the addition of CaCl_2_ or EGTA, and are given in nmol NAD(P)H min^-1^ mg^-1^ ±SD for two independent mitochondrial preparations, each being measured 3 times. The traces shown are from one representative set of measurements. Similar effects over time were seen in all replicates. The inset plot shows the half-life (t_1/2_) of the activity after the EGTA addition ±SD. Significant differences determined by ANOVA is denoted for p<0.05).

In plant cells, the concentration of free Ca^2+^ in the cytosol is strongly fluctuating, displaying upward transient spikes over a low “resting” concentration around 100 nM [40]. External NADH oxidation in *H*. *tuberosus* mitochondria has been shown to be slowly responsive to a decreased Ca^2+^ [39]. We therefore investigated if the NAD(P)H oxidation in potato mitochondria would respond rapidly or slowly to sudden changes in the Ca^2+^ concentration. Under continuous measurement in a dual-wavelength spectrophotometer equipped with rapid stirring, an initial NAD(P)H oxidation rate was measured in the presence of 50 μM EGTA to certify minimal Ca^2+^-concentrations. Surplus amounts of CaCl_2_ and EGTA were then consecutively injected into the medium (Fig 4). Rapid Ca^2+^-induced activation was observed for both NADH and NADPH oxidation at both pH 6.8 and pH 7.2 (half maximum stimulation occurred after less than 5 s). Also, at pH 6.8 both NADH and NADPH oxidation responded rapidly to EGTA addition. However, at pH 7.2 NADH and NADPH oxidation deviated. It took substantially longer time for the NADH oxidation to reach steady state after the EGTA-induced decrease in free Ca^2+^ (half-maximum inhibition reached after 35 s), whereas for NADPH oxidation half-maximum inhibition occurred in 5 s (Fig 4). This indicates that StNDB1 is rapidly responsive to changes in cytosolic Ca^2+^ concentrations, whereas the external NADH DHs turn off slowly in response to a decrease in cytosolic Ca^2+^.

## Discussion

### A single external NADPH DH is measured in potato mitochondria

We have investigated NADPH oxidation by overexpressed AtNDB1 with DcQ as final electron acceptor [32] and in potato mitochondria using DcQ and oxygen. To certify that the measurements in mitochondria only involved the external NADPH DH, inner membrane intactness for NADH was determined by a novel latency test based on previously published results [36]. Here, the external NADH DH is inhibited by EGTA, and the effect of the membrane channel-forming peptide Alm on the oxidation of NADH via internal complex I and type II NADH DHs is determined. The results clearly showed a very high integrity of the purified mitochondria, ascertaining that only external NAD(P)H dehydrogenases are measured and consistent with the previous report [36] regarding NADH oxidation activities and latencies. Inner membrane integrity for NAD(H) has generally been estimated based on the effect of a detergent on the activity of a soluble citric acid cycle enzyme like malate DH or fumarase [41]. However, such latency tests are not directly valid for estimating the inner membrane integrity because the mitochondrial outer surface may bind cytosolic isoforms and enzymes released from broken mitochondria. Latencies calculated from published data [36] on malate DH ± Triton X-100 (81%) and internal NADH DH ± Alm (89%) consistently suggest that malate DH latency underestimates the inner membrane integrity, whereas the NADH DH ± Alm assay should allow an improved estimate. It should be noted that activity of external NADH DHs would artefactually raise the non-latent activity, leading to an underestimated integrity. Thus, the NADH DH±Alm-based latency test demands that external NADH DHs can be inhibited. This can be done with EGTA in mitochondria from some plant materials (e.g. potato tubers, pea leaf and sugarbeet roots) but not from cold-treated sugarbeet roots or *A*. *thaliana* seedlings, which contain Ca^2+^-independent external NADH DHs [27,42,43].

Three families of type II NAD(P)H DHs are present in plant mitochondria, and among these only the NDB-proteins are external [9,44,45]. The orthologues AtNDB1 and StNDB1 specifically oxidise NADPH whereas AtNDB2 and AtNDB4 oxidise only NADH [23,32]. AtNDB3 was predicted to oxidise NADH [23]. Among the three NDB homologues found in the potato genome (S2 Fig), only StNDB1 display the motif for NADPH [23] whereas StNDB2 and StNDB3 proteins are similar to the AtNDB2-4 group of NADH DHs. Consistently, across a wide pH range all external NADPH oxidation (Ca^2+^-dependent and -independent) in *N*. *sylvestris* was co-suppressed with the StNDB1 (Fig 1). Thus, potato mitochondrial oxidation assays should only involve the StNDB1 for NADPH, but may involve two NDB proteins for NADH. A pattern with one NDB1 orthologue and multiple paralogues of NDB2-4-type has also been observed in rice [26] indicating that the existence of a single NDB1 isoform may be common in plants.

### Activation of external NAD(P)H DHs

Cytosolic NAD(P)H is known to be oxidised by plant mitochondria via separate NADH- and NADPH-specific DHs as evidenced by differential induction, tissue dependence and inhibitor sensitivities of the activities [25,27,46]. In *A*. *thaliana* mitochondria, AtNDB1 and AtNDB2 contain canonical EF hand motifs [47] that mediate high-affinity binding of Ca^2+^ [32]. In each protein, the canonical EF-hand motif resides upstream of an EF hand-like sequence in an EF hand domain, which is absent in NDA and NDC proteins and in yeast and bacterial homologues [32,44]. A similar inserted domain is present in the Ca^2+^-independent AtNDB4 NADH dehydrogenase, but this domain contains protein segments that deviate from EF hand motifs in several positions [32]. Potato contains two genes of NDB2/NDB4-type and is also expected to contain both Ca^2+^-dependent and -independent NADH DHs since NADH oxidation is variably dependent on Ca^2+^ in mitochondria from different tissues [27]. In *H*. *tuberosus* mitochondria, external NADH oxidation has a K_0.5_(Ca^2+^) of 0.2–1 μM at pH 7.2 [28,29]. The K_0.5_(Ca^2+^) for NADPH oxidation has not been reported, but maximum activity has been denoted to be reached at 1 μM Ca^2+^ for *H*. *tuberosus* [29] and 30 μM added Ca^2+^ for potato cell and durum wheat mitochondria [48].

Using precisely measured Ca/EGTA buffers and potato tuber mitochondria we determined the K_0.5_(Ca^2+^) for NADH oxidation to oxygen to be around 1 μM at both pH 6.8 and 7.5. However, the K_0.5_(Ca^2+^) for NADPH oxidation was substantially higher and also pH-dependent, ranging from 4–6 μM Ca^2+^ at pH 6.8 to 80–120 μM Ca^2+^ at pH 7.5, with pH 7.2 being intermediate (Figs 2 and 3). A pH-dependent change in Ca^2+^-binding to EF-hand proteins has been reported for the bovine and salmonid cardiac troponin C, where K_0.5_(Ca^2+^) was 15–20 times higher at pH 7.0 than at pH 7.6 [49]. Thus, the pH effect on K_0.5_(Ca^2+^) for NADPH oxidation may be due to a direct pH effect on the NDB1 protein, changing its Ca^2+^ affinity. Using DcQ as electron acceptor in AtNDB1 and StNDB1, we observed similar pH dependences as for oxygen reduction via the native ubiquinone, further indicating that pH and Ca^2+^ affected the NDB1 enzyme directly. However, K_0.5_(Ca^2+^) with DcQ was approximately 4 times lower than with oxygen across the pH range. These observations suggest that the binding of Ca^2+^ to the EF-hand domain in StNDB1 affects binding of the natural ubiquinone and DcQ differentially. Enzyme kinetics of the type II NADH DH in *Yarrowia lipolytica* (NDH2e) have suggested a ping-pong mechanisms and NADH and DcQ interacting at overlapping sites [50]. A close proximity of the NADH and quinone active sites was also observed in the structure of the homodimeric *S*. *cerevisiae* NDI1p type II NADH DH [51]. However, in a separate investigation, the same protein was found to contain two separate quinone-binding sites, where one is mediating electron transport to the other [52]. If the latter case is correct and valid for the plant NDB1 homologue, the membrane-associated native ubiquinone and the soluble DcQ analogue may bind to separate sites and differentially affect Ca^2+^ binding to the EF hand domain. The plant NDB1 EF-hand domain is attached to the NAD(P)H DH domain as an extension of a surface-exposed loop, which is juxtaposed to the dimerisation surface in the *S*. *cerevisiae* NDI1p [51,52], and therefore should not pose a steric constraint for dimerisation (results not shown). Oligomeric native sizes of type II NAD(P)H DHs (NDA and NDB proteins) have been reported for plants [53,54].

Several soluble mammalian Ca^2+^-binding proteins, including recoverin, calmodulin, and guanylate cyclase-activating proteins interact also with Mg^2+^, affecting the Ca^2+^ binding [55]. In our measurements, however, the presence and absence of 2.5 mM Mg^2+^ had no effect on NADPH oxidation nor K_0.5_(Ca^2+^) by potato mitochondria or AtNDB1 (Fig 3). This indicates that the Ca^2+^ binding sites regulating NADPH oxidation are specific to Ca^2+^.

### External NAD(P)H DH activities under in vivo conditions

It has previously been suggested that the external NADPH oxidation in plants can function by reoxidising NADPH derived from chloroplast export of surplus reductant [56] and/or by activating the pentose phosphate pathway and citrate metabolism to support biosynthesis and rapid cell growth [57]. The latter demands that the NADPH DH activity is able to modify the cytosolic NADPH/NADP^+^-ratio. Experiments with *N*. *sylvestris* expressing StNDB1 have shown that the levels of external NADPH DH activity is inversely related to the NADPH/NADP^+^-ratio in leaves at lower growth light and in stems at higher growth light [19]. Consistently, RNAi inhibition of the AtNDB1 gene lead to elevated NADPH/NADP^+^-ratios in *A*. *thaliana* leaves under relatively low growth light [21]. This shows that the NDB1 enzyme is active in vivo in these organs and under these growth conditions and indicates that the cytosolic pH and Ca^2+^ levels allow NDB1 activity under these conditions.

Cytosolic pH is regulated in most organisms to be kept in a narrow range around 7.2–7.5 and has likewise been concluded to be slightly alkaline in plants under normal conditions, i.e. 7.0–7.5 [58], 7.2–7.5 [59] or 7.4 [60], but can increase under carbon starvation [61]. When plant cells are under abiotic stress (e.g. in hypoxic environments) the cytoplasmic pH can decrease to at least 6.8, yet cytosolic acidification has also been observed in response to changes in light intensity and symbiotic or pathogenic microorganisms [59]. Regarding NADPH oxidation, a cytosolic acidification is expected to activate NDB1 by decreasing the demand for Ca^2+^ (Fig 3). During stress caused by hypoxia, cold and heat, the cellular NADPH/NADP^+^-ratio increases [62] and an activation of NDB1 may ameliorate overreduction of the cellular NADPH pool, avoiding reductive stress [63]. Especially under hypoxia, the elevated NADPH/NADP^+^-ratio is accompanied by cytosolic acidification which thus could signal for increased NADPH oxidation by activating NDB1.

NDB1 is oriented towards the intermembrane space and the outer membrane is generally considered to be permeable to small molecules [64]. Nevertheless, the pH gradient across the outer membrane have been estimated to be 0.1–0.5 and 0.7 in isolated rat liver mitochondria and in mitochondria in human ECV304 cells, respectively [65,66]. For the assay on potato mitochondria, a pH gradient may have resided across the outer membrane equalising this effect, whereas the AtNDB1 was measured in *E*. *coli* membranes and the enzyme may therefore be more activated by a lower local pH when residing in mitochondria.

Ca^2+^ is a common second messenger in eukaryotic signalling, working via control of both genes and enzyme activities [67]. In a “resting” plant cell, the Ca^2+^ concentration is in the order of magnitude of 0.1 μM, but abiotic and biotic stimuli induces transient Ca^2+^ spikes, which are further connected to Ca^2+^ sensor proteins that regulate transcriptional and metabolic responses [2]. Such spikes can reach cytosolic Ca^2+^ concentrations of at least 5 μM in response to external stress [3]. In mammalian cells, histamin-induced Ca^2+^ elevations will also lower the local cytosolic pH by 0.1 units [68], but it is not known if this also influences the mitochondrial intermembrane space. Ca^2+^ signals can affect plant mitochondria via the outer membrane pores and inner membrane Ca^2+^ transport systems, to directly or indirectly regulate mitochondrial enzymes [64].

Whereas the K_0.5_(Ca^2+^) for external NADH oxidation was consistent with previous reports and also independent of pH, we observed that surprisingly high Ca^2+^ concentrations were needed to activate the NDB1 enzymes, and especially at a pH above neutral (Figs 2 and 3). This indicates that the NDB1 NADPH DH can only be active during simultaneously decreased pH and elevated Ca^2+^, as compared to a “resting cell”. The slow inactivation of NADH oxidation by a decreased Ca^2+^ concentration indicates that recurrent spikes of elevated Ca^2+^ will lead to a permanently activated NADH oxidation. However, the fast inactivation of NADPH oxidation by decreased Ca^2+^ indicates that the NADPH oxidation would more strictly follow the fluctuations in cytosolic Ca^2+^. This is consistent with observed fluctuations of the same period for NAD(P)H fluorescence and Ca^2+^ in pollen tubes, though it is not known if the pool of fluctuating NAD(P)H was intra- or extramitochondrial [69]. The dependence of NDB1 on rapidly changing Ca^2+^ levels does not exclude a role for NDB1 and changes in cytosolic NADPH/NADP^+^-ratio in growth. For example, addition of auxin to isolated protoplasts from leaves of wheat seedlings induced a rapid increase in the cytosolic Ca^2+^ concentration [70].

In conclusion, we observe that the regulation of the external NADPH DH in plant mitochondria is substantially different from that of the homologous NADH DHs. The pH-dependent demand for high Ca^2+^ concentrations by the NADPH DH and the rapid adjustment to Ca^2+^ variations indicate that the NADPH DH is highly responsive to changes in the cytosolic conditions (e.g. due to signalling or metabolic shifts). In contrast, NADH oxidation is more constitutively activated, being dependent on lower Ca^2+^ concentrations and slowly inactivated by a decrease in Ca^2+^.

## Supporting Information

S1 Fig Calcium electrode response curve. The figure shows an example of the final calculated pCa values for free Ca^2+^ concentrations of the different Ca/EGTA-buffered media aliquots plotted against the potential determined for each aliquot using the Ca^2+^ electrode. For very low Ca^2+^ concentrations (pCa > 7), the response was not linear. One representative experiment using Medium 1 at pH 7.2 is shown. The apparent Ca/EGTA stability constant in this experiment was 5.742 10^6^ M^-1^.
(TIF)

S2 Fig Alignment of StNDB1 and AtNDB1 to other NDB proteins in the same species. Proteins were aligned using Clustal Omega (http://www.ebi.ac.uk) and used BoxShade (http://www.ch.embnet.org) for shading background according to conservation. Sequence numbering is according to the full-length protein sequences. GenBank accession numbers are as follows: StNDB1 (gi:5734587), AtNDB1 (gi:18417151), StNDB2 (gi:565360770), StNDB3 (gi:565382402), AtNDB2 (gi:18412775), AtNDB3 (gi:240256027), AtNDB4 (gi:15225428). The NAD(P)H binding motif and positions deviating between NADPH and NADH DHs are denoted by ** (Michalecka et al., 2004). EF-hand and EF-hand-like motifs in AtNDB1 and StNDB1 are denoted as previously described (Geisler *et al*., 2007). The latter deviates in the NADH DH-types of homologues.
(TIF)
