# Supplementary figures and images for: The Ca2+-Regulation of the Mitochondrial External NADPH Dehydrogenase in Plants Is Controlled by Cytosolic pH
Source: PLoS One. 2015 Sep 28;10(9):e0139224. doi: 10.1371/journal.pone.0139224 (PMC4587368; doi:10.1371/journal.pone.0139224)

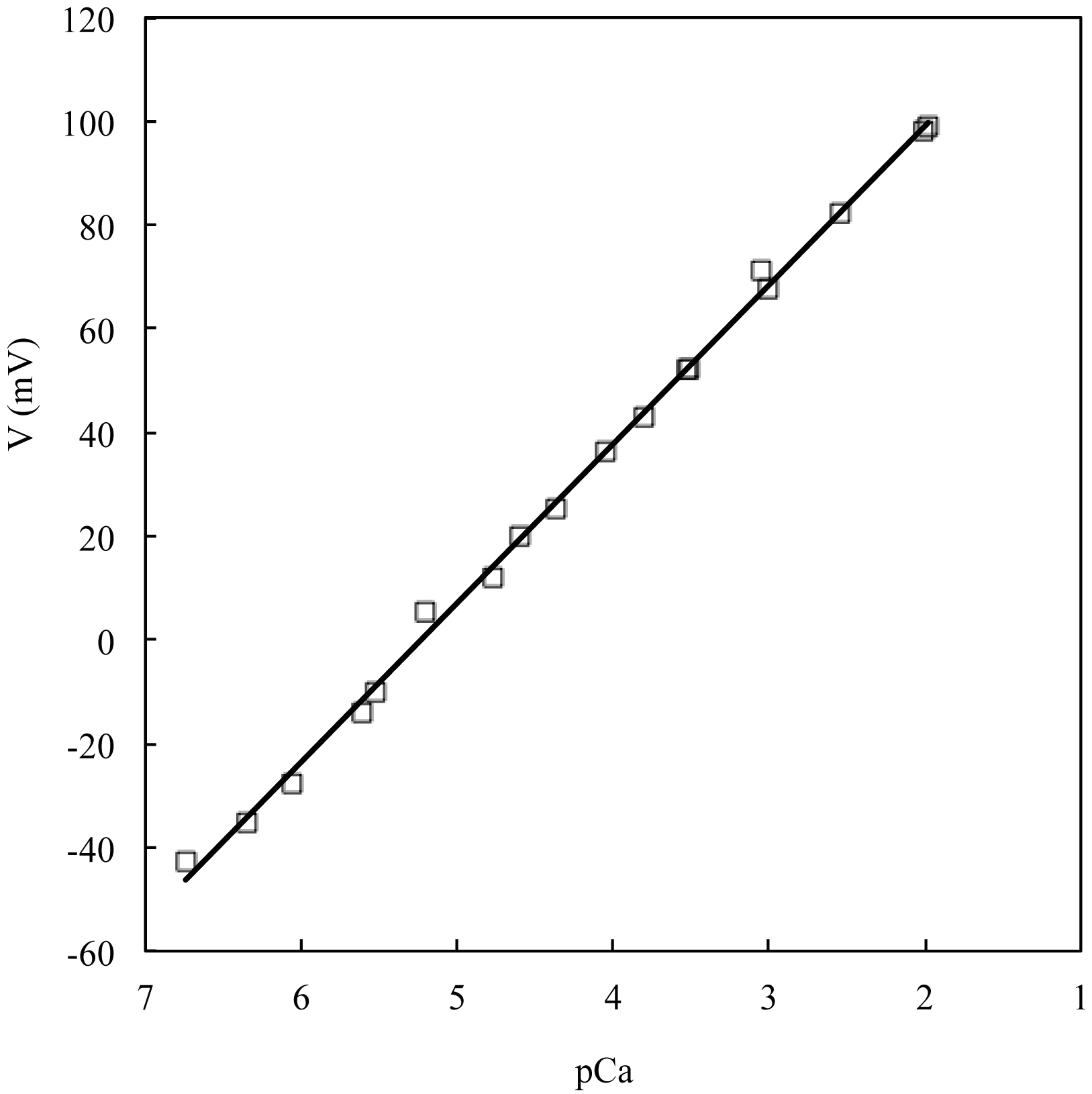

Supplement: S1 Fig — The figure shows an example of the final calculated pCa values for free Ca2+ concentrations of the different Ca/EGTA-buffered media aliquots plotted against the potential determined for each aliquot using the Ca2+ electrode. For very low Ca2+ concentrations (pCa > 7), the response was not linear. One representative experiment using Medium 1 at pH 7.2 is shown. The apparent Ca/EGTA stability constant in this experiment was 5.742 106 M-1. (TIF) [file pone.0139224.s001.tif]

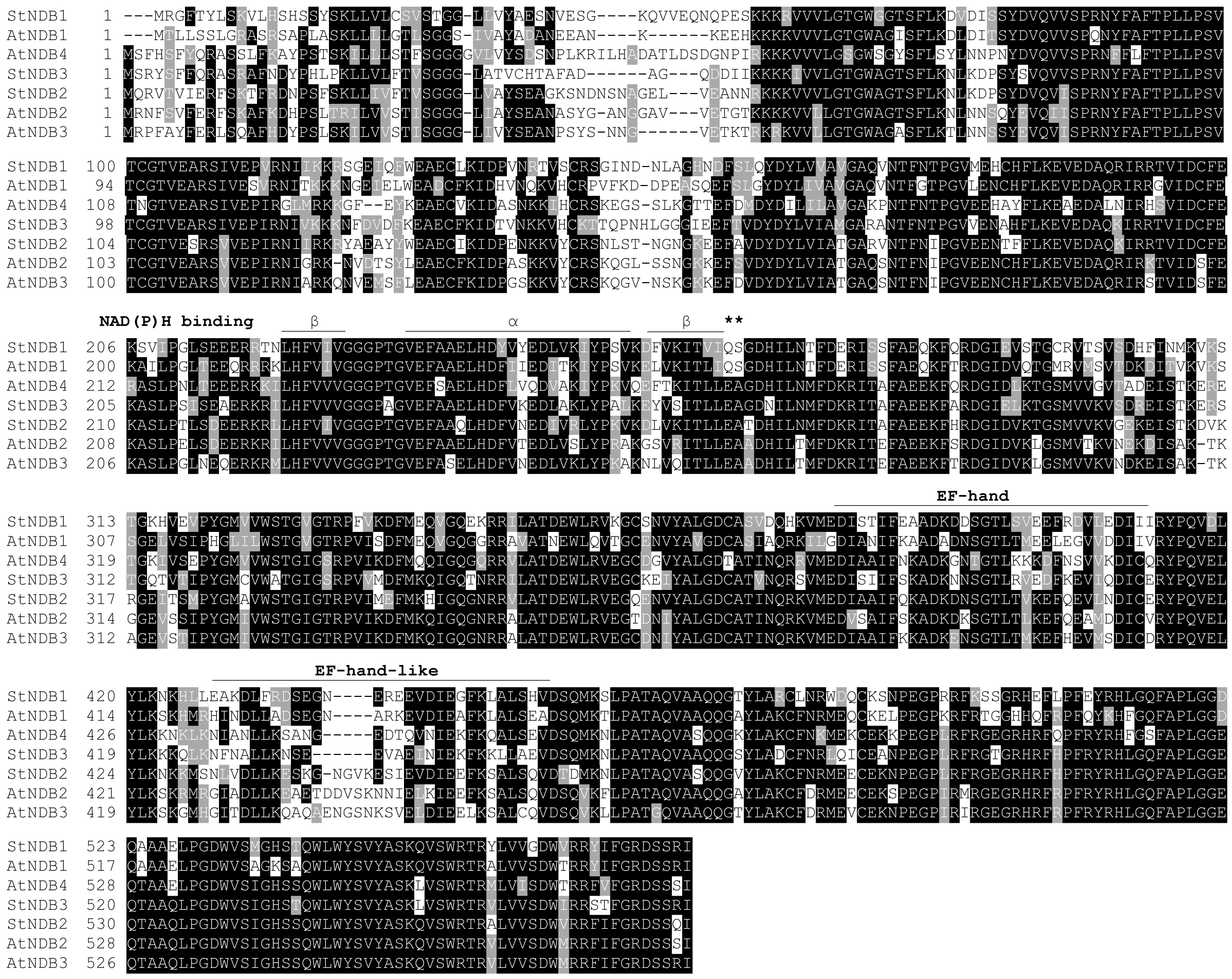

Supplement: S2 Fig — Proteins were aligned using Clustal Omega (http://www.ebi.ac.uk) and used BoxShade (http://www.ch.embnet.org) for shading background according to conservation. Sequence numbering is according to the full-length protein sequences. GenBank accession numbers are as follows: StNDB1 (gi:5734587), AtNDB1 (gi:18417151), StNDB2 (gi:565360770), StNDB3 (gi:565382402), AtNDB2 (gi:18412775), AtNDB3 (gi:240256027), AtNDB4 (gi:15225428). The NAD(P)H binding motif and positions deviating between NADPH and NADH DHs are denoted by ** (Michalecka et al., 2004). EF-hand and EF-hand-like motifs in AtNDB1 and StNDB1 are denoted as previously described (Geisler et al., 2007). The latter deviates in the NADH DH-types of homologues. (TIF) [file pone.0139224.s002.tif]
